# Clinical Parameters in Osteoporosis Patients Supplemented With PMA-Zeolite at the End of 5-Year Double-Blinded Clinical Trial

**DOI:** 10.3389/fmed.2022.870962

**Published:** 2022-06-27

**Authors:** Sandra Kraljević Pavelić, Dalibor Krpan, Marta Žuvić, Sandra Eisenwagen, Krešimir Pavelić

**Affiliations:** ^1^Faculty of Health Studies, University of Rijeka, Rijeka, Croatia; ^2^Polyclinic “K – centre”, for Internal Medicine, Gynaecology, Radiology, Physical Medicine and Rehabilitation, Zagreb, Croatia; ^3^Department of Biotechnology, University of Rijeka, Rijeka, Croatia; ^4^Panaceo International GmbH, Godersdorf, Austria; ^5^Faculty of Medicine, Juraj Dobrila University of Pula, Pula, Croatia

**Keywords:** clinical trial, osteoporosis, PMA-zeolite, clinoptilolite, bone fracture, surrogate bone metabolic markers

## Abstract

Osteoporosis is among the most common pathologies. Associated complications in osteoporotic patients, in particular hip fractures and vertebral fractures, cause disabilities and significant quality of life deterioration. Standard treatment of osteoporosis, based on pharmacotherapy does still not yield adequate results, and the problem of osteoporosis remains incompletely solved. Additionally, adverse drug events and fractures after long-termed pharmacotherapy pose additional challenges within designing a proper therapy regimen. Improved clinical approach and new synergistic treatment modalities are consequently still needed. The rationale of the presented study was accordingly, to expand our preclinical animal study on human patients with osteoporosis, based on positive effects on bones observed in animals with osteopenia treated with PMA-zeolite. We specifically monitored effects of PMA-zeolite on the bone quality parameters, fracture risk and quality of life in a cohort of initially recruited 100 osteoporosis patients during a follow-up period of 5 years within a randomized, placebo-controlled and double blinded clinical study (TOP study). Obtained results provide evidence on the PMA-zeolite positive effects on the bone strength of osteoporotic patients as the risk of fractures was significantly decreased in PMA-zeolite-treated patients with respect to time before entering the study (*p* = 0.002). Statistical evidence point also to positive bone changes in the 5-years TOP study course as evidenced through osteocalcin and beta-cross laps values showing a prevalence of the bone-formation process (*p* < 0.05). BMD values were not significantly affected after the 5-years follow-up in PMA-zeolite-treated patients in comparison with the Placebo group. Results support the initial expectations based on our previously published preclinical studies on clinoptilolite product PMA-zeolite in animals that could be a new therapeutic option in osteoporosis patients.

## Introduction

Osteoporosis is acknowledged as a common medical problem and a public health issue. This pathology is associated with osteoporosis complications that cause disabilities and significant deterioration of the quality of life due to compromised bone strength and increased risk of fractures. Accordingly, complications associated with these diseases, in particular hip fractures and vertebral fractures, are the major cause of disabilities and significant deterioration of the quality of life, induce large financial burdens for the patients, their families and the whole society (1). According to the consensus reached in 2001 by NIH Consensus Development Panel on Osteoporosis, osteoporosis is defined as “*a skeletal disorder characterized by compromised bone strength and micro-architecture predisposing a person to an increased risk of fracture”*. Accordingly, in the clinical practice and research, osteoporosis stays for bone quality degradation and bone weakness, resulting in bone fractures. Particularly, the vertebral fractures are in focus of osteoporosis complications reflecting decreased bone strength and quality (2). Fractures amid minor trauma are a consequence of poor bone strength, and therefore the best evidence of osteoporosis. Bone strength primarily depends on the quality of collagen and bone microarchitecture, more than on bone mass or bone mineral density. In osteoporosis however, significant decrease of BMD occurs as well due to metabolic changes in the bone. Accordingly, the bone reabsorption underlined by osteoclast activity prevails over bone formation and osteoblast activity leading to a reduced bone mass and increased risk of fracture. These processes are the main targets of osteoporosis therapy and drugs. For example, bisphosphonates alendronate, risedronate, ibandronate, pamidronate, and zoledronic acid induce osteoclast apoptosis which may be monitored in patients by the surrogate marker of bone reabsorption, the beta-cross laps (amino- and carboxyl-terminal breakdown products of type 1 collagen in serum and urine) (1, 3). Moreover, monoclonal antibodies act on signaling pathways relevant for the bone reabsorption, i.e., denosumab acts against RANK ligand (RANKL), a regulator of osteoclast differentiation, activation and survival (4, 5). Despite various available pharmacological treatments, the problem of osteoporosis is not yet solved nor decreased. In addition, available treatments often exhibit a variety of unwanted side effects and existing real life data do not support their clear anti-fracture effects in a long-term, i.e., more than 1 year (6, 7).

Consequently, development of new therapeutic possibilities, especially adjuvant therapeutic approaches, is desirable. In evaluation of the therapeutic effect of any potential treatment for osteoporosis, the best evidence of a successful treatment would be the measurement of improved bone strength parameters. Still, no diagnostic tool or method to measure the exact bone strength exists or is fully adequate at the moment. Surrogate parameters such as bone mineral density (BMD) and bone markers of bone reabsorption may be for example, monitored within a clinical trial course, yielding data on the bone metabolism status. Also, the parameters on the quality of bone collagen, and bone microarchitecture, crucial for bone strength evaluation, may remain inconclusive. So far, most of the clinical studies of osteoporosis treatments, evaluated fracture risk as a parameter of therapeutic effect, but comparing the incidence of fractures regardless of the incidence of trauma or fall down (1, 8). One possible approach for example, to assess the clear reduction of fracture risk in a treatment regimen may be within a long-term follow-up where the probability of potential fallings is increased.

The rationale of the presented study was accordingly, an additional exploration on human subjects of previously obtained positive effects of PMA-zeolite on bones of animals with osteopenia. Having in mind the requirements in testing of a potential intervention in osteoporosis patients, we monitored the effects of PMA-zeolite on bone quality parameters, fracture risk and quality of life in the follow-up period of 5 years within a randomized, placebo-controlled and double blinded study (TOP study). The follow-up period of 5-years allowed for clear evaluation of the potential anti-fracture risk in patients with documented fracture-risk trauma events.

## Materials and Methods

### Zeolite Material

Clinoptilolite material PMA-zeolite was provided by Panaceo International GmbH, Austria. PMA (Panaceo Micro Activation)-zeolite (patent WO2018/100178A1) is a certified European medical device as per European Union directive 93/42/EEC and subjected to required toxicology tests performed according to the OECD and ISO guidelines. The PMA-zeolite medical device physical and chemical properties have been described previously (9).

### Clinical Study “Treatment of Osteoporosis by Panaceo, TOP”

Previous data from the preclinical and first year of the TOP study were previously published in Kraljević Pavelić et al. (10). Herein, we present the summarized data of the whole randomized, placebo-controlled and double blinded TOP study (First year of the study: ClinicalTrials.gov Identifier: NCT03901989, https://clinicaltrials.gov/ct2/show/NCT03901989?term=zeolite&draw=2&rank=5; Treatment of Osteoporosis - second to fifth year (Pear Control): ClinicalTrials.gov Identifier: NCT05178719, https://clinicaltrials.gov/ct2/show/NCT05178719?term=zeolite&draw=2&rank=6). The total exploratory 5-years TOP study lasted in total for 5 years and aimed to measure long-term PMA-zeolite effects on the bones of osteoporosis patients supplemented with PMA-zeolite for a period up to 4 or 5 years (48 or 60 months, respectively). The total number of patients enrolled in the TOP study was 100 and the initial number of PMA-zeolite-supplemented subjects in the first year was *n* = 50. The placebo-group in the first year of the study (*n* = 50) received microcrystalline cellulose powder similar in appearance to PMA-zeolite as already used in previous studies (11, 12). The placebo subjects were given PMA-zeolite (group PMA-zeolite-supplemented subjects) after the end of first year until the end of the study (end of fifth year). After the 5th year, the total number of subjects supplemented with PMA-zeolite was 55 (sample description and drop-out reasons are given in Supplementary Material). The PMA-zeolite-treated patients received 9 g/day of PMA-zeolite for 48 months (patients enrolled within the first year as Placebo and afterwards supplemented with PMA-zeolite for 4 years) or 60 months (PMA-treated patients group supplemented with PMA-zeolite from the beginning of the study up to 5 years).

Inclusion criteria:

- main inclusion criteria used to recruit patients was the lowered BMD value (T score −2,5 and/or lower) with no prior therapy. A total of 100 male and female osteoporotic patients (Croatian Caucasians) from 56 to 74 years of age were recruited accordingly.

Exclusion criteria:

- other severe diseases such as cancer, autoimmune disease, chronic renal failure
- female subjects who were pregnant were excluded from the study
- secondary osteoporosis

All patients recruited in the TOP study were supplemented with Vitamin D3 at the dosage of 800 IU per day. The reasons for exclusion of patients during the TOP study course are given in details in the Supplementary Material. Briefly, withdrawal reasons included lack of compliance with the supplementation protocol, minor side effects or change of residence. To ensure compliance, the subjects were contacted every 3 months for a complete check-up and health monitoring and to receive motivation to remain compliant with the protocol.

**Figure 1:**
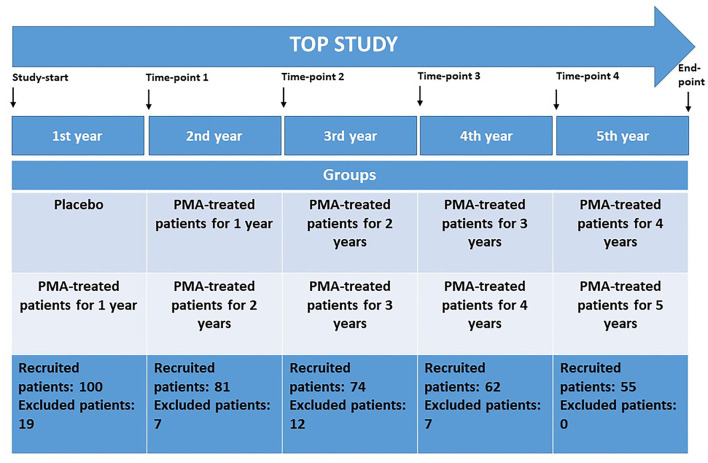
The TOP study design and corresponding study time measurement points at the end of each study year. Study groups and recruited patients at the beginning of each study year are given. Placebo-control in the first study year; PMA-zeolite treated patients—groups receiving PMA-zeolite supplementation for 1 year up to 5 years.

TOP study time points are as follows (Figure 1):

- Time point 0—beginning of the TOP study
- Time point 1—end of first study year (1Y)
- Time point 2—end of second study year (2Y)
- Time point 3—end of third study year (3Y)
- Time point 4—end of fourth study year (4Y)
- Time point 5—end of fifth study year (5Y)

The TOP study groups are as follows (Figure 1):

- Placebo—patients receiving placebo from time point 0 to time point 1
- “PMA-treated patients 1Y”—patients treated with PMA-zeolite from time point 0 to time point 1 or treated with PMA-zeolite from time point 1 to time point 2
- PMA-treated patients 2Y—patients treated with PMA-zeolite from time point 0 to time point 2 or treated with PMA-zeolite from time point 1 to time point 3
- “PMA-treated patients 3Y”—patients treated with PMA-zeolite from time point 0 to time point 3 or treated with PMA-zeolite from time point 1 to time point 4
- “PMA-treated patients 4Y”—patients treated with PMA-zeolite from time point 0 to time point 4 or treated with PMA-zeolite from time point 1 to time point 5
- “PMA-treated patients 5Y”—patients treated with PMA-zeolite from time point 0 to time point 5
- “PMA-treated patients final”—patients treated with PMA-zeolite from time point 1 to time point 5 and patients treated with PMA-zeolite from time point 0 to time point 5.

### Osteoporosis Status Assessment

Subjects were monitored at the start of the study and at the end of each study year. The surrogate markers of bone status were measured as follows: BMD, changes in levels of osteocalcin and beta-crosslaps CTx assay. Densitometry was performed by standard Hologic Discovery DXA system (Marlborough, Massachusetts, USA). Precisely, the BMD was assessed by bone densitometry of lumbar vertebrae (L1–L4) and the femoral neck. Calcifications and deformities due to bone fractures were excluded to avoid potential skewing of data. In addition, subjects provided additional data including the frequency of falling and the occurrence of fractures before entering the study (period of 1–5 years prior to entering the TOP study and during the TOP study course). Osteocalcin and beta-cross laps were assessed in the accredited laboratory of the Polyclinics Breyer (Croatia) by use of electrochemiluminescence immunoassay (eCLIA). Specifically, for osteocalcin measurements Elecsys N-MID Osteokalcin eCLIA platform (Roche, Germany) was employed on Modular Analytics (immunochemistry modules cobas e602, cobas e601 and E170) (Roche, Germany). For beta-cross laps measurements Elecsys Beta-CrossLaps/serum eCLIA platform (Roche, Germany) was employed on Modular Analytics (immunochemistry modules cobas e602, cobas e601 and E170) (Roche, Germany). At the conclusion of the study, subjects were evaluated for general health status which was compared to that at the start of the study. Quality of life has been assessed by presence of bone fractures before entering the study and during the study, occurrence of fall accidents, and the level of pain (on Visual Analog Scale, VAS) at the beginning and at the end of the study. Additionally, subjective assessment of overall health condition was assessed by participants at the end of the study. Participants assessed their condition on the scale from 1 to 5, where numbers indicate: 1—much worse than before entering the study, 2—worse than before entering the study, 3—unchanged, 4—better than before entering the study, 5—much better than before entering the study. Side effects were monitored and documented through close clinical examination by the physician during the whole study course and this included regular standard blood test monitoring as well.

### Randomization

Subjects were randomized into blocks of four and sequentially numbered within two groups receiving either PMA-zeolite-clinoptilolite or placebo (microcrystalline cellulose powder) in the first study year (Research Randomizer (Version 4.0) [Computer software]. **(author?)** (27). Retrieved on June 22, 2013, from http://www.randomizer.org/). In the first year (first 12 months of the TOP study), the patients received either PMA-zeolite-clinoptilolite or placebo powder which are similar in appearance. Starting from the second TOP study year all subjects received boxes containing PMA-zeolite-clinoptilolite until the end of the TOP study (in total for 5 years). All subjects received 9 g/day PMA-zeolite-clinoptilolite powder (Panaceo International GmbH, Villach, Austria). All subjects were instructed to take 1 spoon of the powder dissolved in a glass of water three times daily: with breakfast, with lunch, and with dinner for a period of a total of up to 5 years (until the end of the TOP study). Subjects were furthermore instructed to maintain their daily diet and lifestyle throughout the study. No osteoporosis drugs and supplements except for Vitamin D3 were allowed within the TOP study. To ensure compliance, the subjects were contacted every 3 months for a complete check-up and health monitoring and to receive motivation to remain compliant with the protocol.

### Statistical Methods

Clinical trial data were analyzed using Dell Statistica, version 12 (2015). The variables are presented as frequencies or percentages and compared using the Pearson chi-square test or Fisher exact test, where appropriate. Normally distributed continuous variables (distribution tested with Kolmogorov-Smirnov test) are presented as means with standard deviation, otherwise as median with interquartile range (IQR). Comparisons of variables in two independent groups (Placebo, PMA-treated) were done using the parametric *t*-test or non-parametric Mann Whitney U test. Statistical significance was determined at the level of 0.05.

## Results

### Bone Quality Parameters

Summarized results of the surrogate bone metabolic markers data from the years 1–5 of the TOP study are presented in the Supplementary Table 2. The subgroup analysis of diabetic patients is also given in the Supplementary Table 3. The entering point for Placebo comparisons was the time point 1 (end of the first study year) as it represents a clinically meaningful point for comparisons with PMA-treated patients groups. The group “PMA-treated patients—final” should be carefully taken into consideration as it gathers all patients supplemented with PMA-zeolite either for 4 or 5 years, respectively until the end of the TOP study. Statistically relevant comparisons of bone surrogate metabolic markers (differences at *p* < 0.05) in patients for each tested time-point are presented in Table 1.

**Table 1:** Statistically relevant differences for analyzed bone surrogate metabolic markers (BMD, osteocalcin and beta-cross laps) values in patients according to the tested time-points.

**Bone remodelling value**	**Comparison**	***p***	**Relative Δ value %**
**Placebo at time point 1 vs. PMA-treated patients 1Y at the end of the second study year**.		
BMD g/cm^2^ mean ± SD	0.662 ± 0.108 vs. 0.652 ± 0.097	0.950	−1.5%
**Osteocalcin ng/ml, mean** **± SD**	**23.9** **±6.9 vs. 29.1** **±12.3**	0.001^*^	**+21.6%**
Beta-cross laps ng/ml, mean ± SD	0.39 ± 0.14 vs. 0.37 ± 0.16	0.355	−3.9%
**Placebo at time point 1 vs. PMA-treated patients 2Y at the end of the second study year**.		
BMD g/cm^2^ mean ± SD	0.662 ± 0.108 vs. 0.681 ± 0.133	0.121	+2.9%
**Osteocalcin ng/ml, mean** **±SD**	**23.9** **±6.9 vs. 28.8** **±10.2**	0.001^*^	**+20.2%**
Beta-cross laps ng/ml, mean ± SD	0.39 ± 0.14 vs. 0.39 ± 0.20	0.954	+1.8%
**Placebo at time point 1 vs. PMA-treated patients 1Y+2Y at the end of the second study year**.		
BMD g/cm^2^ mean ± SD	0.662 ± 0.108 vs. 0.666 ± 0.116	0.928	+0.6%
**Osteocalcin ng/ml, mean** **±SD**	**23.9** **±6.9 vs. 28.9** **±11.2**	0.011^*^	**+20.9%**
Beta-cross laps ng/ml, mean ± SD	0.39 ± 0.14 vs. 0.38 ± 0.18	0.915	−1.0%
**Placebo at time point 1 vs. PMA-treated patients 3Y** **+** **4y at the end of the fourth study year**.		
BMD g/cm^2^ mean ± SD	0.662 ± 0.108 vs. 0.635 ± 0.109	0.235	−6.1%
Osteocalcin ng/ml, mean ± SD	23.9 ± 6.9 vs. 26.7 ± 8.1	0.080	+13.1%
**Beta-cross laps ng/ml, mean** **±SD**	**0.39** **±0.14 vs. 0.33** **±0.13**	0.034^*^	**−3.6%**
**Placebo at time point 1 vs. PMA-treated patients—final at the end of the TOP study**		
BMD g/cm^2^ mean ± SD	0.662 ± 0.108 vs. 0.642 ± 0.110	0.498	−6.3%
**Osteocalcin ng/ml, mean** **±SD**	**23.9** **±6.9 vs. 28.4** **±6.9**	0.002^*^	**+21.1%**
**beta-cross laps ng/ml, mean** **±SD**	**0.39** **±0.14 vs. 0.32** **±0.13**	0.014^*^	**−4.3%**
**Placebo at time point 1 vs. PMA-treated patients 4Y at the end of the fourth study year**.		
BMD g/cm^2^ mean ± SD	0.662 ± 0.108 vs. 0.648 ± 0.105	0.605	−6.3%
Osteocalcin ng/ml, mean ± SD	23.9 ± 6.9 vs. 26.1 ± 9.1	0.269	+13.2%
**Beta-cross laps ng/ml, mean** **±SD**	**0.39** **±0.14 vs. 0.32** **±0.12**	0.040^*^	**−1.9%**
**Placebo at time point 1 vs. PMA-treated patients 4Y at the end of the TOP study**.		
BMD g/cm^2^ mean ± SD	0.662 ± 0.108 vs. 0.634 ± 0.115	0.328	−7.8%
**Osteocalcin ng/ml, mean** **±SD**	**23.9** **±6.9 vs. 29.8** **±6.8**	0.001^*^	**+21.2%**
Beta-cross laps ng/ml, mean ± SD	0.39 ± 0.14 vs. 0.35 ± 0.16	0.288	−4.4%
**PMA-treated patients 1Y at time point 1 vs. PMA-treated patients 5Y at time point 5**		
BMD g/cm^2^ mean ± SD	0.639 ± 0.096 vs. 0.652 ± 0.107	0.616	−3.7%
Osteocalcin ng/ml, mean ± SD	24.0 ± 6.8 vs. 26.8 ± 6.9	0.115	−6.0%
**Beta-cross laps ng/ml, mean** **±SD**	**0.40** **±0.14 vs. 0.29** **±0.10**	0.001^*^	**−4.2%**
**PMA-treated patients 1Y at time point 1 vs. PMA-treated patients 4Y at the end of the TOP study**.		
BMD g/cm^2^ mean ± SD	0.639 ± 0.096 vs. 0.634 ± 0.115	0.848	−0.23%
**Osteocalcin ng/ml, mean** **±SD**	**24.0** **±6.8 vs. 29.8** **±6.8**	0.001^*^	**+27.6%**
Beta-cross laps ng/ml, mean ± SD	0.40 ± 0.14 vs. 0.35 ± 0.16	0.181	−9.2%

*Results are presented as relative* Δ *value %*.

* *Statistically relevant changes at p < 0.05 are denoted in bold and with an asterisk*.

Osteocalcin values were statistically increased in PMA-zeolite-treated patients in the relative Δ value ranging from +20.2% to +27.6% in comparison with Placebo or PMA-treated patients 1Y time point 1 (Placebo time point 1 vs. PMA-treated patients 1Y time point 2, *p* = 0.001; Placebo time point 1 vs. PMA-treated patients 2Y time point 2, *p* = 0,001; Placebo time point 1 vs. PMA-treated patients 1Y+2Y time point 2, *p* = 0.011; Placebo time point 1 vs. PMA-treated patients—final time point 5, *p* = 0.002; Placebo time point 1 vs. PMA-treated patients 4Y time point 5, *p* = 0.001; PMA-treated patients 1Y time point 1 vs. PMA-treated patients 4Y time point 5). Moreover, beta-cross laps were statistically decreased in PMA-zeolite-treated patients in the relative Δ value ranging from −1.9 to −9.2% in comparison with Placebo or PMA-treated patients 1Y time point 1 (Placebo time point 1 vs. PMA-treated patients 3Y+4Y time point 4, *p* = 0.034; Placebo time point 1 vs. PMA-treated patients—final time point 5, *p* = 0.014; Placebo time point 1 vs. PMA-treated patients 4Y time point 4, *p* = 0.040; PMA-treated patients 1Y time point 1 vs. PMA-treated patients 5Y time point 5, *p* = 0.001).

**Figure 2:**
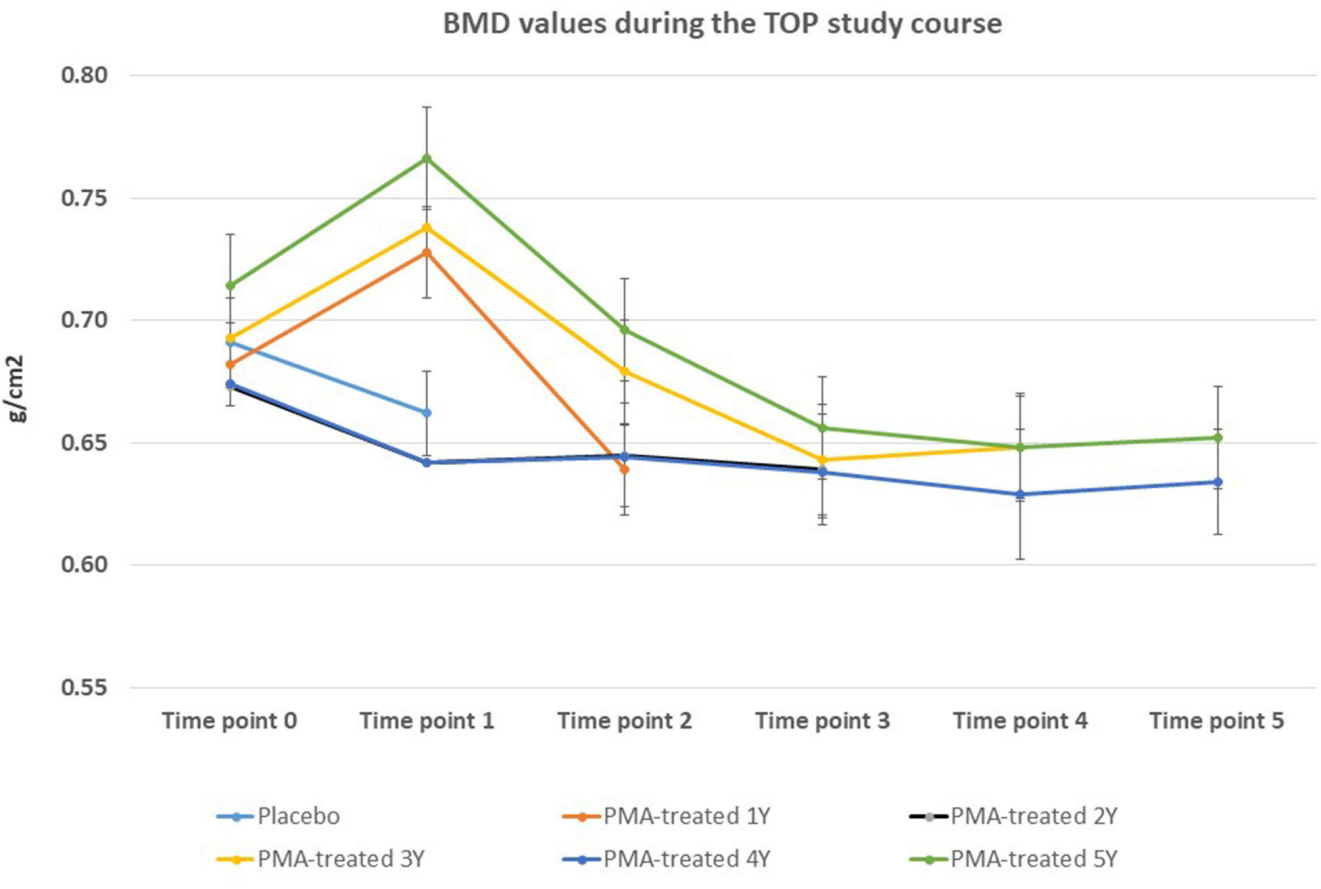
Changes in BMD values from the beginning of the study (time point 0) until the end of study (time point 5) for Placebo and PMA-treated patients. A separate representation for the patients enrolled as Placebo group at time point 0 (later treated with PMA-zeolite from time point 1–5) and enrolled patients treated with PMA-zeolite from the beginning of the study (time point 0–5) is given in the Supplementary Figure 1.

**Figure 3:**
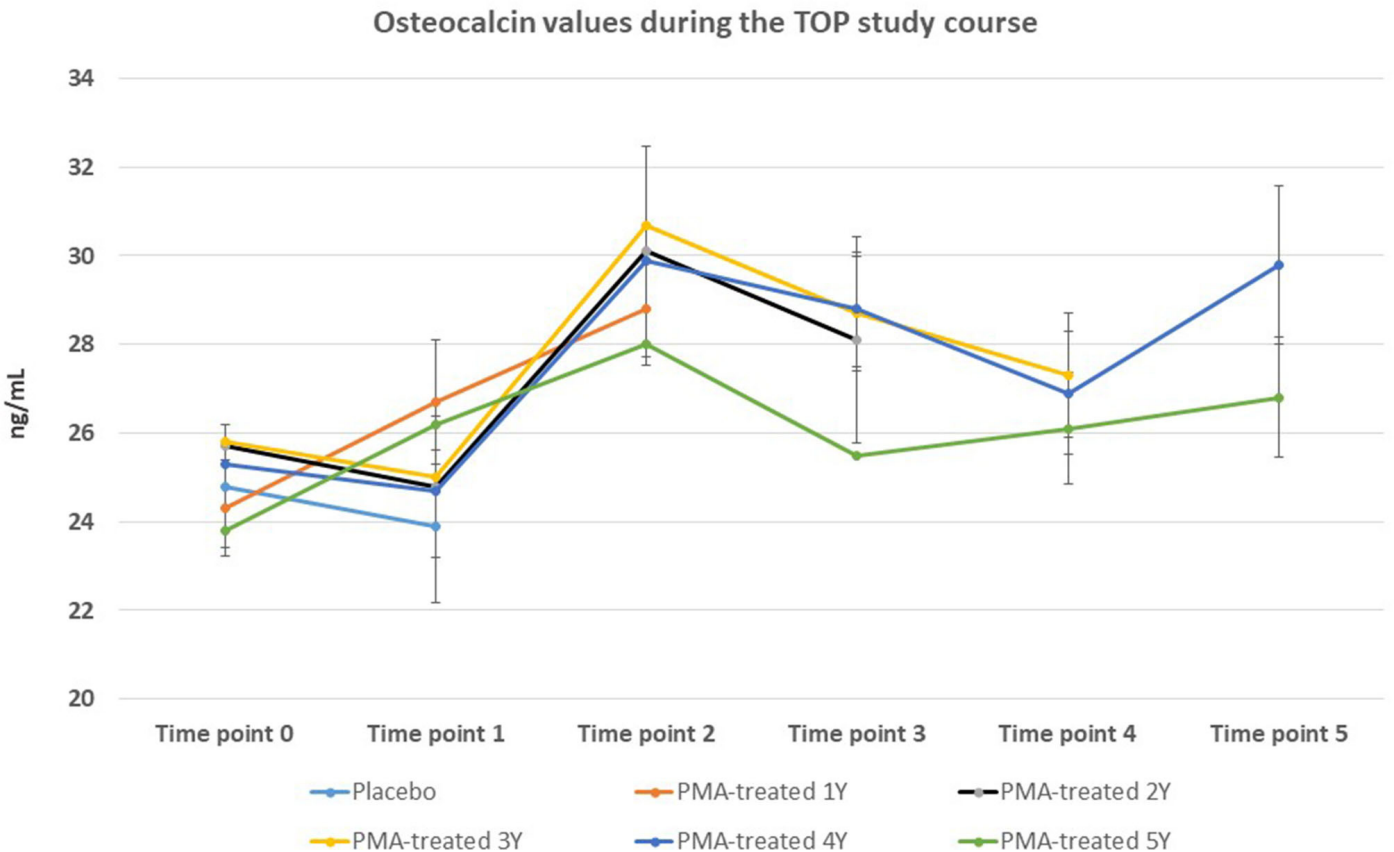
Changes in osteoclacin values from the beginning of the study (time point 0) until the end of study (time point 5) for Placebo and PMA-treated patients. A separate representation for the patients enrolled as Placebo group at time point 0 (later treated with PMA-zeolite from time point 1–5) and enrolled patients treated with PMA-zeolite from the beginning of the study (time point 0–5) is given in the Supplementary Figure 1.

**Figure 4:**
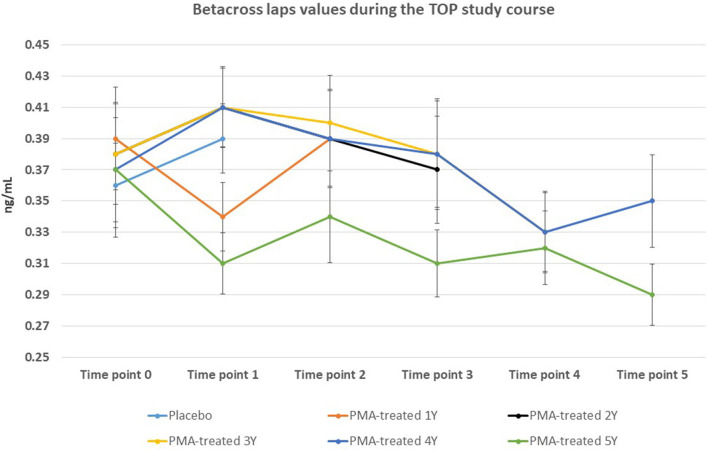
Changes in beta-cross laps values from the beginning of the study (time point 0) until the end of study (time point 5) for Placebo and PMA-treated patients. A separate representation for the patients enrolled as Placebo group at time point 0 (later treated with PMA-zeolite from time point 1–5) and enrolled patients treated with PMA-zeolite from the beginning of the study (time point 0–5) is given in the Supplementary Figure 1.

The BMD values were not significantly affected within the 5-year course of PMA-zeolite-supplementation which is not surprising knowing that bone density is not the only relevant bone metabolic and fitness status *marker*. Moreover, over-increased ossification is not a desired osteoporosis therapy outcome as it may contrary decrease the bone strength. Importantly, we observed that the bone metabolism surrogate markers, osteocalcin and beta cross laps showed a statistically relevant changes toward bone remodeling processes. Osteocalcin values were accordingly statistically increased in PMA-zeolite-treated patients in comparison with Placebo (Table 1), as well as the beta cross laps were statistically decreased in PMA-zeolite-treated patients in comparison with Placebo (Table 1). These changes are graphically depicted in Figures 2–4.

At last, side effects were monitored throughout the study and only 6 patients reported side effects as constipation problems (5 patients) or as transient hot flashes and tension in breasts (1 patient) (Supplementary Material). The monitored standard blood values were in the reference range throughout the study for all patients. Compliance to the therapeutic intervention (PMA-zeolite) was generally good and a total of 19% of PMA-zeolite treated patients in the 5-year TOP study course were excluded due to non-compliance. This percentage was 6% for the Placebo group during the first TOP study year.

### Quality of Life Parameters

Quality of life has been assessed by presence of bone fractures before entering the study and during the study, occurrence of fall accidents, and the level of pain (on Visual Analogue Scale, VAS) at the beginning and at the end of the study. Additionally, subjective assessment of overall health condition was assessed at the end of the study. Results are summarized in Table 2.

**Table 2:** Analysis of values relevant for the quality of life in Placebo at the end of the TOP study first year vs. PMA-treated patients—final at the end of the TOP study.

	**Placebo at the end of first year** **(*N* = 40)**	**PMA-treated patients—final at the end of the study (*N* = 55)**	***p***
**Bone fractures** before entering the study*, n (%)*	9 (22.5%)	9 (29.0%)	0.589
**Bone fractures** during the study*, n (%)*	1 (2.5%)	0 (0.0%)	0.999
***p***	0.007^*^	0.002^*^	
**Fall accidents during the study** *, n (%)*	0 (0.0)	24 (43.6%)	<0.001^*^
^*^VAS at the beginning, *median (IQR)*	3 (1–5)	2 (1–7)	0.547
**VAS at the end**, *median (IQR)*	2 (1–4)	0 (0–1)	<0.001^*^
***p***	0.007^*^	<0.001^*^	
**ΔVAS**, *median (IQR)*	0 (0–0)	−3 (−5 to −2)	<0.001^*^
**Subjective assessment of overall health condition at the end of the study**, *median (IQR)*	3 (3,4)	5 (4,5)	<0.001^*^

* VAS, visual analogue scale; IQR, interquartile range. Statistically relevant differences at p > 0.05 are denoted in bold and with an asterisk (^*^).

The obtained results of the fracture numbers for a 5-years follow-up period (from the beginning of the study at time point 0 to the end of study at time point 5) in correlation with falls, showed that the number of fall accidents was high during the study course (*N* = 24) resulting in a statistically lower number of fractures (*p* = 0.002) in comparison with the number of fractures documented before entrance into the TOP study. This result is in correlation with surrogate bone metabolic markers changes during the study course that pointed to a decreased bone extracellular matrix degradation visible as statically relevant decrease of beta-cross laps in the PMA-treated patients 5Y group (*p* = 0.028). The level of pain (VAS) was also significantly decreased during the course of TOP study (<0.001) and decreased for 3 points at the end of the TOP study. The subjective assessment of overall health condition at the end of the TOP study also increased in the fifth study year for 2 points (<0.001^*^).

## Discussion

Fracture amid minor trauma is still a highly frequent osteoporosis complication that results in serious deterioration of quality of life and causes permanent disability in significant percentage of patients (13). Therefore, prevention of fractures is the major goal of the treatment of osteoporosis. Fractures amid minor trauma in osteoporotic patients result from poor bone strength. As the bone strength primarily depends on the quality of collagen and bone architecture, the BMD parameter does not give conclusive data on the bone strength and quality. Accordingly, the results from the TOP study presented herein comprise data on the bone metabolism parameters osteocalcin and beta-cross laps and bone density BMD. In addition, long-term follow-up data on fracture risk for the period of 5-years has been collected. The TOP study was designed indeed, as a long-term study that followed specifically the incidence of fracture in patients who had significant trauma. The bone strength depends mostly on the quality of collagen (osteoid) which gives a flexibility and microarchitecture, that cannot be measured directly *in vivo* (14). In our previous study by using the osteopenia rat model over a 16-week PMA-zeolite treatment, PMA-zeolite supplementation showed significant improvements in bone density markers and bone architecture and morphometric characteristics (10). These data were used as a rationale for the herein presented clinical study on human subjects diagnosed with osteoporosis. We specifically monitored effects of PMA-zeolite on the bone quality parameters, fracture risk and quality of life in a cohort of initially recruited 100 osteoporosis patients during a follow-up period of 5 years within a randomized, placebo-controlled and double blinded clinical study (TOP study). The TOP study provided additional evidence on the PMA-zeolite positive effects on the bone strength of osteoporotic patients as the risk of fractures, as a consequence of all documented fall accidents (a total of 24 severe fall accidents were documented during the TOP study), was significantly decreased in PMA-zeolite-treated patients with respect to time before entering the study (*p* = 0.002). Statistical evidence on changes toward bone remodeling process in the 5-years TOP study course was also evidenced through osteocalcin and beta-cross laps values showing a prevalence of the bone-formation process (Table 1). Importantly, the BMD values were not significantly affected after the 5-years follow-up in PMA-zeolite-treated patients in comparison with Placebo. This seem to point to a balanced remodeling process important for the bone homeostasis, that is often missing in a long-term pharmacological approach to osteoporosis. Indeed, current pharmacological interventions used for osteoporosis management induce either inhibition of bone resorption or stimulate bone formation (15). For example, it has been shown that bisphosphonates can increase bone mineral density (BMD) but concomitantly have a negative effect on the flexibility of bone that finally increases the fracture risk (15, 16). In addition, drugs that induce bone formation processes, i.e., PTH analogs and sclerostin inhibitors, studied as the alternative to bisphosphonates for osteoporosis treatment, also remain questionable in terms of cost-effectiveness and adverse effects in a therapeutic regimen longer than 2-years (15). Still, osteoporosis treatment options for reversing bone loss and promoting bone regeneration in the same intervention are currently limited (17). This is why therapeutic approaches in osteoporotic patients are often based on sequential and combinational use of currently available drugs from the groups of anabolic and anti-resorptive drugs with the aim to promote a balanced bone turnover. In line with this, the PMA-zeolite treatment may be an interesting option in designing of a patient-tailored long-term osteoporosis treatment regimen where a balanced homeostasis of bone remodeling process is a desired therapeutic outcome. Therapy compliance to the osteoporosis pharmacological therapy remains a challenge as well, due to side effects (18, 19) or small benefits for the patient's quality of life (19, 20). In the TOP study, the compliance to PMA-zeolite supplementation was 81% and interesting data was obtained within the quality of life assessment as well. Indeed, the estimate of overall health condition at the end of the TOP study was significantly better in the group of osteoporotic patients supplemented with PMA-zeolite for 5 years (*p* = 0.001). Importantly, no severe side-effects or worsening of symptoms were observed in PMA-zeolite-supplemented subjects during the TOP study course.

The mechanisms of action of the observed positive effects on the bone strength are hypothesized to be attributable to the physical-chemical properties of the PMA-zeolite as an inorganic material. Furthermore, this material releases soluble silica forms from its surface that may have positive effects on the bone (21, 22). Proven detoxifying properties of the clinoptilolite materials including PMA-zeolite may also improve the overall health and bone status (23, 24). At last, local effects in the intestine as the primary site of PMA-zeolite action, i.e., on the microbiota status, as well as corresponding anti-inflammatory and immunomodulatory effects, are likely to contribute to the bone health as well (25, 26).

In conclusion, the PMA-zeolite intervention may be evaluated as a valuable approach in therapy of osteoporotic patients. In our first publication (10) covering the first TOP study year results, an increase in bone mineral density, a significant reduction in pain (*p* < 0.05). Upon completion of the 5-year TOP study period the data provided more conclusive results, as a significant decrease of the fracture risk in PMA-zeolite-treated patients with respect to time before entering the study (*p* = 0.002) was observed at the end of the TOP study. Statistical evidence on changes toward a balanced bone remodeling process in the 5-years TOP study course was also evidenced in PMA-zeolite-treated patients through osteocalcin and beta-cross laps values showing a prevalence of the bone-formation process (*p* < 0.05) without significant changes in the BMD values. In particular, ostecalcin values were significantly increased in PMA-treated groups in comparison with Placebo at *p* < 0.05 after the end of second, fourth and fifth TOP study years. The beta-cross laps were decreased in PMA-treated groups in comparison with Placebo at *p* < 0.05 after the end of fourth and fifth Top study years. These trends were observed in PMA-treated patients at all study time-points as well. The BMD values were not statistically changed in PMA-treated groups. At last, improved overall quality of life in osteoporotic patients enrolled in the explorative TOP-study and treated with PMA-zeolite was observed and evidenced as an increase for 2 points (*p* < 0.001) in the fifth TOP study year. The intervention also proved generally safe for consumption in human subjects treated with 9 g of 100% PMA-zeolite powder per day for 5 years as no standard blood value changes or substantial side effects and severe side effects were observed during the TOP study course. Even though, presented data already prove the positive impact of PMA-zeolite over a 5-year intake period, further, preferably multi-centric studies are desirable in order to additionally substantiate the positive benefit to risk ratio documented herein.

### Limitations Regarding the Generalization of the Findings

Presented data refer to a specially micronized natural clinoptilolite, referred to as PMA-zeolite. A generalization of the findings presented in this paper to other clinoptilolite materials that may have different structural and surface properties. Previously published data demonstrated that the micronization technology (Panaceo Micro Activation—PMA, patent WO2018/100178A1) is changing the biophysical properties of the natural clinoptilolite (9). Based on the interesting findings of the TOP study, larger, preferably multi-centric studies, are desirable to additionally substantiate the positive benefit to risk ratio documented herein.

## Data Availability Statement

The original contributions presented in the study are included in the article/Supplementary Material, further inquiries can be directed to the corresponding author/s.

## Ethics Statement

Clinical trial was conducted according to the guidelines of the Declaration of Helsinki for Research on Human Subjects 1989 and an informed consent was signed by all enrolled subjects as per usual recommendations in vigour in the Republic of Croatia. The osteoporosis TOP study (https://clinicaltrials.gov/, NCT03901989) was approved by the Ethical committee of the University of Rijeka, Department of Biotechnology on 3rd April, 2014 (reference number 001-2013). An Ethical approval for extension of the study in the fourth and fifth year of the TOP study was also obtained by the Polyclinic K-centre Ethical committee (permission from 5th February 2018).

## Author Contributions

SK drafted the manuscript, analyzed and interpreted the data, and performed literature search. DK supervised and conducted clinical part of the study. MŽ performed statistical analysis of obtained data. SE performed technical support to the clinical trial registration and organization. KP and DK designed the clinical trials. KP led the project and performed final manuscript critical revision. All authors participated in literature search and manuscript preparation and approved the final manuscript content.

## Funding

The Clinical trial was funded by a research grant from Panaceo International GmbH, Villach, Austria (Osteoporosis TOP (Treatment of osteoporosis by Panaceo) clinical Studies TOP1-TOP5). The funder was not involved in the study design, collection, analysis, interpretation of data, the writing of this article or the decision to submit it for publication.

## Conflict of Interest

SK and KP are independent scientific advisors of Panaceo International GmbH, Austria. SE is employed at Panaceo International Gmbh, Austria. The remaining authors declare that the research was conducted in the absence of any commercial or financial relationships that could be construed as a potential conflict of interest.

## Publisher's Note

All claims expressed in this article are solely those of the authors and do not necessarily represent those of their affiliated organizations, or those of the publisher, the editors and the reviewers. Any product that may be evaluated in this article, or claim that may be made by its manufacturer, is not guaranteed or endorsed by the publisher.

## References

[1] Yusuf AA Cummings SR Watts NB Feudjo MT Sprafka JM Zhou J. Real-world effectiveness of osteoporosis therapies for fracture reduction in post-menopausal women. Arch Osteoporos. (2018) 13:33. 10.1007/s11657-018-0439-3 29564735 PMC5862911

[2] Lorentzon M Cummings SR. Osteoporosis: the evolution of a diagnosis. J Int Med. (2015) 277:650–61. 10.1111/joim.12369 25832448

[3] Drake MT Clarke BL Khosla S. Bisphosphonates: mechanism of action and role in clinical practice. Mayo Clin Proc. (2008) 83:1032–45. 10.4065/83.9.1032 18775204 PMC2667901

[4] Ringe JD Farahmand P. Improved real-life adherence of 6-monthly denosumab injections due to positive feedback based on rapid 6-months BMD increase and good safety profile. Rheumatol Int. (2014) 34:727–32. 10.1007/s00296-012-2663-2 23334374

[5] Zhang N Zhang Z-K Yu Y Zhuo Z Zhang G Zhang B-T. Pros and cons of denosumab treatment for osteoporosis and implication for RANKL *Aptamer Ther Front Cell Dev Biol*. (2020) 8:325. 10.3389/fcell.2020.00325 32478071 PMC7240042

[6] Skjødt MK Frost M Abrahamsen B. Side effects of drugs for osteoporosis and metastatic bone disease. Br J Clin Pharmacol. (2019) 85:1063–71. 10.1111/bcp.13759 30192026 PMC6533454

[7] Tu KN Lie JD Wan CKV Cameron M Austel AG Nguyen NK. Osteoporosis: a review of treatment options. P T. (2018) 43:92–104. 29386866 PMC5768298

[8] Lin SL Hung M-C Chang S-F Tsuang F-Y Chang JZ-C Sun J-S. Efficacy and safety of postmenopausal osteoporosis treatments: a systematic review and network meta-analysis of randomized controlled trials. J Clin Med. (2021) 10:3043. 10.3390/jcm10143043 34300210 PMC8305263

[9] Kraljević Pavelić S Micek V Filošević A Gumbarević D Žurga P Bulog A. Novel, oxygenated clinoptilolite material efficiently removes aluminium from aluminium chloride-intoxicated rats *in vivo*. Micropor Mesopor Mat. (2017) 249:146–56. 10.1016/j.micromeso.2017.04.062

[10] Kraljević Pavelić S Micek V Bobinac D Bazdulj E Gianoncelli A Krpan D. Treatment of osteoporosis with a modified zeolite shows beneficial effects in an osteoporotic rat model and a human clinical trial. Exp Biol Med. (2020) 246:529–37. 10.1177/1535370220968752 33183068 PMC7930600

[11] Vitale MG Barbato C Crispo A Habetswalner F DeMartino BM Riccardi F. ZeOxaNMulti trial: a randomized, double-blinded, placebo-controlled trial of oral PMA-zeolite to prevent chemotherapy-induced side effects, in particular, peripheral neuropathy. Molecules. (2020) 25:2297. 10.3390/molecules25102297 32414185 PMC7288011

[12] Lamprecht M Bogner S Steunbauer K Schuetz B. Effects of zeolite supplementation on parameters of intestinal barrier integrity, inflammation, redoxbiology and performance in aerobically trained subjects. J Int Soc Sports Nutr. (2015) 12:40. 10.1186/s12970-015-0101-z 26500463 PMC4617723

[13] Krpan D Kullich W. Nuclear magnetic resonance therapy (MBST) in the treatment of osteoporosis. Case report study. Clin Cases Miner Bone Metab. (2017) 14:235–8. 10.11138/ccmbm/2017.14.1.235 29263740 PMC5726216

[14] Hart NH Nimphinos S Rantalainen T Ireland A Siafarikas A Newton RU. Mechanical basis of bone strength: influence of bone material, bone structure and muscle action. J Musculoskelet Neuronal Interact. (2017) 3:114–39. 28860414 PMC5601257

[15] Föger-Samwald U Dovjak P Azizi-Semrad U Kerschan-Schindl K Pietschmann P. Osteoporosis: pathophysiology and therapeutic options. EXCLI J. (2020) 19:1017–37. 10.17179/excli2020-2591 32788914 PMC7415937

[16] Jensen PR Andersen TL Chavassieux P Roux JP Delaisse JM. Bisphosphonates impair the onset of bone formation at remodeling sites. Bone. (2021) 145:115850. 10.1016/j.bone.2021.115850 33465485

[17] Russow G Jahn D Appelt J Märdian S Tsitsilonis S Keller J. Anabolic therapies in osteoporosis and bone regeneration. Int J Mol Sci. (2018) 20:83. 10.3390/ijms20010083 30587780 PMC6337474

[18] Li S-S He S-H Xie P-Y Li W Zhang X-X Tian-Fang L. Recent progresses in the treatment of osteoporosis. Front Pharmacol. (2021) 12:717065. 10.3389/fphar.2021.717065 34366868 PMC8339209

[19] Li H Xiao Z Quarles LD Li W. Osteoporosis: mechanism, molecular target and current status on drug development. Curr Med Chem. (2021) 28:1489–507. 10.2174/0929867327666200330142432 32223730 PMC7665836

[20] Fuggle NR Cooper C Harvey NC Al-Daghri N Brandi ML Bruyere O. Assessment of cardiovascular safety of anti-osteoporosis drugs. Drugs. (2020) 80:1537–52. 10.1007/s40265-020-01364-2 32725307 PMC7536167

[21] Munjas Jurkić L Cepanec I Kraljević Pavelić S Pavelić K. Biological and therapeutic effects of ortho-silicic acid and some ortho-silicic acid-releasing compounds: new perspectives for therapy. Nutr Metabol. (2013) 10:2. 10.1186/1743-7075-10-2 23298332 PMC3546016

[22] Ha S-W Viggeswarapu M Habib MM Beck GR. Bioactive effects of silica nanoparticles on bone cells are size, surface, and composition dependent. Acta Biomater. (2018) 82:184–96. 10.1016/j.actbio.2018.10.018 30326276 PMC10321369

[23] Kraljević Pavelić S Simović Medica S Gumbarević D Filošević A PrŽulj N Pavelić K. Critical review on zeolite clinoptilolite safety and medical applications *in vivo*. Front Pharmacol. (2018) 9:1350. 10.3389/fphar.2018.01350 30538633 PMC6277462

[24] Serati-Nouri H Jafari A Roshangar L Dadashpour M Pilehvar-Soltanahmadi Y Zarghami N. Biomedical applications of zeolite-based materials: a review. Mater Sci Eng C Mater Biol Appl. (2020) 116:111225. 10.1016/j.msec.2020.111225 32806312

[25] Lyu W Jia H Deng C Yamada S Kato H. Zeolite-containing mixture alleviates microbial dysbiosis in dextran sodium sulfate-induced colitis in mice. Food Sci Nutr. (2020) 9:772–80. 10.1002/fsn3.2042 33598162 PMC7866626

[26] Quach D Britton RA. Gut microbiota and bone health. Adv Exp Med Biol. (2017) 1033:47–58. 10.1007/978-3-319-66653-2_4 29101651

[27] Urbaniak GC Plous S. Research Randomizer (Version 4.0). (2013). Available online at: http://www.randomizer.org/

