# Supplementary material for: Clinical Parameters in Osteoporosis Patients Supplemented With PMA-Zeolite at the End of 5-Year Double-Blinded Clinical Trial
Source: Front Med (Lausanne). 2022 Jun 27;9:870962. doi: 10.3389/fmed.2022.870962 (PMC9272402; doi:10.3389/fmed.2022.870962)
Supplement: Supplementary file 1 [file Data_Sheet_1.docx]

**Supplementary material for the paper:**

**Clinical Parameters in Osteoporosis Patients Supplemented With PMA-Zeolite at the End of 5-Year**

**Double-Blinded Clinical Trial**

**Sandra Kraljević Pavelić^1^, Dalibor Krpan^2^, Marta Žuvić^3^, Sandra Eisenwagen^4^, Krešimir Pavelić^5^**

^1^ University of Rijeka, Faculty of Health Studies, Viktora Cara Emina 5, 51000 Rijeka, Croatia

^2^ Polyclinic “K – center”, for internal medicine, gynecology, radiology, physical medicine and rehabilitation, Vrbik 8a, 10 000 Zagreb, Croatia

^3^ University of Rijeka, Department of Biotechnology, Radmile Matejčić 2, 51000 Rijeka, Croatia

^4^ Panaceo International GmbH, Finkensteinerstr. 5, A-9585, Godersdorf, Austria

^5^ Faculty of Medicine, Juraj Dobrila University of Pula, Zagrebačka 30, 52100 Pula, Croatia

**Correspondence:** Prof. Sandra Kraljević Pavelić, University of Rijeka, Faculty of Health Studies, Viktora Cara Emina 5, 51000 Rijeka, Croatia, e-mail: [sandrakp@uniri.hr](mailto:sandrakp@uniri.hr)

**Table of Contents**

[1. Sample description and drop-out of patients during the TOP study 5-year course 3](#_Toc106259489)

[2. Bone quality parameters measured during the TOP study course 6](#_Toc106259490)

[3. Analysis of diabetic patients’ subgroup enrolled in the TOP study 12](#_Toc106259491)

# Sample description and drop-out of patients during the TOP study 5-year course

Supplementary Table 1. Demographic and clinical anamnesis (diabetes) data of patients’ groups in the TOP study.

|  | **First study year** | | | | | **Second study year** | | | | | **Third study year** | | | | |
| --- | --- | --- | --- | --- | --- | --- | --- | --- | --- | --- | --- | --- | --- | --- | --- |
| **Group** | **PMA-supplemented patients** | | **Placebo** | | **p** | **PMA-supplemented patients 1Y** | | **PMA-supplemented patients 2Y** | | **p** | **PMA-supplemented patients 2Y** | | **PMA-supplemented patients 3Y** | | **p** |
| N | 41 | | 40 | | - | 37 | | 37 | | - | 31 | | 31 | | - |
| **Age** / years, *mean ± SD* | 66 ± 8 | | 65 ± 10 | | 0.927 | 65 ± 8 | | 65 ± 11 | | 0.999 | 66 ± 10 | | 64 ± 8 | | 0.360 |
| **Gender** – female, *n (%)* | 38 (92.7%) | | 38 (95.0%) | | 0.665 | 34 (91.9%) | | 35 (94.6%) | | 0.679 | 29 (93.5%) | | 28 (90.3%) | | 0.641 |
| **Diabetes**, *n (%)* | 9 (21.9%) | | 8 (20.0%) | | 0.829 | 8 (21.6%) | | 5 (13.5%) | | 0.543 | 5 (16.1%) | | 8 (25.8%) | | 0.349 |
|  | | **TOP 4** | | | | | | | **TOP 5** | | | | | | |
| **Group** | | **PMA-supplemented patients 3Y** | | **PMA-supplemented patients 4Y** | | | **p** | | **PMA-supplemented patients 4Y** | | | **PMA-supplemented patients 5Y** | | **p** | |
| N | | 30 | | 31 | | | - | | 29 | | | 26 | |  | |
| **Age** / years, *mean ± SD* | | 67 ± 9 | | 64 ± 8 | | | 0.174 | | 67 ± 10 | | | 63 ± 8 | | 0.110 | |
| **Gender** – female, *n (%)* | | 28  (93.3%) | | 28  (90.3%) | | | 0.668 | | 27  (93.1%) | | | 23  (88.5%) | | 0.550 | |
| **Diabetes**, *n (%)* | | 5  (16.7%) | | 8  (25.8%) | | | 0.384 | | 5  (17.2%) | | | 7  (26.9%) | | 0.385 | |

**EXCLUSION REASONS DURING THE 5-YEARS TOP STUDY COURSE**

**First study year**

Excluded patients: 19 patients (Excluded patients in the PMA-treated group: 9 patients (8 + 1 diabetic), Excluded patients from the Placebo group: 10 patients (8 + 2 diabetics)

One patient was excluded from the study due to side effect. Patient reported that she felt hot flashes and tension in breasts soon after she started to take study drug. After she stopped with medication symptoms disappeared but appeared again when she took a drug again. Although it was not life threatening side effect and despite the physician explanation that the symptoms will most probably disappear soon, she didn’t want to continue. In 5 cases, patients complained on obstipation and asked to be excluded from the study. Two patients have been excluded from the study due to the fact that they have moved out of Croatia for the longer period of time. Eleven patients were excluded from the study because they did not take the study drug in accordance with the protocol.

**Second study year**

Excluded patients: 7 patients

One patient was excluded from the study due to breast cancer which has been found on the routine control. In 5 cases, patients stopped to take a medication without explanation. One patient has been excluded from the study due to the fact that she has moved out of Zagreb and she has not been able to come on regular controls.

**Third to fifth study year**

Excluded patients: 19

Eleven patients have been excluded due to the “violation of the protocol”, because they didn’t take the drug properly. Eight patients were excluded because they were not able coming on the regular controls due they moved to another city.

**Total excluded patients until the end of the 5-year TOP study course: 19 + 7 + 19 = 45**

**Total patients that concluded the TOP study: 55**

# Bone quality parameters measured during the TOP study course

**Supplementary Table 2. Summarized results for the BMD, osteocalcin and betacross laps values within the 5-year TOP study course. Results are presented for time points 0-5 as: BMD in g/cm2 mean ± SD, osteocalcin in ng/ml mean ± SD, and Betacross laps in ng/ml, mean ± SD.**

| **Group** | **N** | **BMD** | | | | | |
| --- | --- | --- | --- | --- | --- | --- | --- |
|  |  | **Time point 0** | **Time point 1** | **Time point 2** | **Time point 3** | **Time point 4** | **Time point 5** |
| **Placebo** | 40 | 0.691 ± 0.116 | 0.662 ± 0.108 |  |  |  |  |
| **PMA-treated 1Y** | 41 | 0.682 ± 0.112 | 0.728 ± 0.119 |  |  |  |  |
| **PMA-treated 1Y** | 37 |  | 0.639 ± 0.096 | 0.652 ± 0.097 |  |  |  |
| **PMA-treated 2Y** | 37 | 0.689 ± 0.108 | 0.670 ± 0.127 | 0.681 ± 0.133 |  |  |  |
| **PMA-treated 1Y + 2Y** | 74 | 0.686 ± 0.113 | 0.655 ± 0.113 | 0.666 ± 0.116 |  |  |  |
| **PMA-treated 2Y** | 31 | 0.673 ± 0.109 | 0.642 ± 0.102 | 0.645 ± 0.102 | 0.639 ± 0.117 |  |  |
| **PMA-treated 3Y** | 31 | 0.692 ± 0.111 | 0.739 ± 0.122 | 0.674 ± 0.137 | 0.643 ± 0.125 |  |  |
| **PMA-treated – 2Y+ 3Y** | 62 | 0.683 ± 0.109 | 0.691 ± 0.122 | 0.692 ± 0.111 | 0.641 ± 0.120 |  |  |
| **PMA-treated 3Y** | 29 | 0.670 ± 0.106 | 0,637 ± 0.099 | 0.642 ± 0.102 | 0.636 ± 0.118 | 0.624 ± 0.113 |  |
| **PMA-treated 4Y** | 26 | 0.693 ±0.111 | 0,738 ± 0.122 | 0.679 ± 0.135 | 0.643 ± 0.125 | 0.648 ± 0.105 |  |
| **PMA-treated 3Y + 4Y** | 55 | 0.682 ± 0.108 | 0,688 ± 0.121 | 0.661 ± 0.120 | 0.640 ± 0.120 | 0.635 ± 0.109 |  |
| **PMA-treated 4Y** | 29 | 0.674 ± 0.108 | 0.642 ± 0.105 | 0.644 ± 0.104 | 0.638 ± 0.119 | 0.629 ± 0.114 | 0.634 ± 0.115 |
| **PMA-treated 5Y** | 26 | 0.714 ± 0.097 | 0.766 ± 0.104 | 0.696 ± 0.133 | 0.656 ± 0.120 | 0.648 ± 0.105 | 0.652 ± 0.107 |
| **PMA-treated – final (4Y+ 5Y)** | 55 | 0.693 ± 0.104 | 0.701 ± 0.121 | 0.669 ± 0.120 | 0.646 ± 0.119 | 0.638 ± 0.109 | 0.642 ± 0.110 |
| **Group** | **N** | **Osteocalcin** | | | | | |
|  |  | **Time point 0** | **Time point 1** | **Time point 2** | **Time point 3** | **Time point 4** | **Time point 5** |
| **Placebo** | 40 | 24.8 ± 8.7 | 23.9 ± 6.9 |  |  |  |  |
| **PMA-treated 1Y** | 41 | 24.3 ± 11.0 | 26.7 ± 8.9 |  |  |  |  |
| **PMA-treated 1Y** | 37 |  | 24.0 ± 6.8 | 29.1 ± 12.3 |  |  |  |
| **PMA-treated 2Y** | 37 | 24.6 ± 11.4 | 27.0 ± 9.1 | 28.8 ± 10.2 |  |  |  |
| **PMA-treated 1Y + 2Y** | 74 | 24.6 ± 10.0 | 25.5 ± 8.1 | 28.9 ± 11.3 |  |  |  |
| **PMA-treated 2Y** | 31 | 25.7 ± 9.2 | 24.8 ± 7.1 | 30.1 ± 13.2 | 28.1 ± 11.0 |  |  |
| **PMA-treated 3Y** | 31 | 22.8 ± 9.4 | 25.8 ± 8.2 | 27.9 ± 10.4 | 25.3 ± 8.7 |  |  |
| **PMA-treated – 2Y+ 3Y** | 62 | 24.3 ± 9.3 | 25.3 ± 7.6 | 29.0 ± 11.8 | 26.7 ± 9.9 |  |  |
| **PMA-treated 3Y** | 29 | 25.8 ± 9.0 | 25.0 ± 6.9 | 30.7 ± 13.0 | 28.7 ± 11.1 | 27.3 ± 7.2 |  |
| **PMA-treated 4Y** | 26 | 24.1 ± 11.2 | 26.6 ± 8.8 | 28.7 ± 10.2 | 25.7 ± 8.7 | 26.1 ± 9.1 |  |
| **PMA-treated 3Y + 4Y** | 55 | 24.9 ± 10.1 | 25.8 ± 7.9 | 29.6 ± 11.6 | 27.2 ± 10.0 | 26.7 ± 8.1 |  |
| **PMA-treated 4Y** | 29 | 25.3 ± 9.4 | 24.7 ± 7.3 | 29.9 ± 13.5 | 28.8 ± 10.9 | 26.9 ± 7.5 | 29.8 ± 6.8 |
| **PMA-treated 5Y** | 26 | 23.8 ± 9.6 | 26.2 ± 8.4 | 28.0 ± 9.6 | 25.5 ± 8.6 | 26.1 ± 9.1 | 26.8 ± 6.9 |
| **PMA-treated – final (4Y+ 5Y)** | 55 | 24.6 ± 9.4 | 25.4 ± 7.8 | 29.0 ± 11.7 | 27.2 ± 9.9 | 26.5 ± 8.2 | 28.4 ± 6.9 |
| **Group** | **N** | **Betacross laps** | | | | | |
|  |  | **Time point 0** | **Time point 1** | **Time point 2** | **Time point 3** | **Time point 4** | **Time point 5** |
| **Placebo** | 40 | 0.36 ± 0.17 | 0.39 ± 0.14 |  |  |  |  |
| **PMA-treated 1Y** | 41 | 0.39 ± 0.21 | 0.34 ± 0.14 |  |  |  |  |
| **PMA-treated 1Y** | 37 |  | 0.40 ± 0.14 | 0.37 ± 0.16 |  |  |  |
| **PMA-treated 2Y** | 37 | 0.40 ± 0.21 | 0.35 ± 0.15 | 0.39 ± 0.20 |  |  |  |
| **PMA-treated 1Y + 2Y** | 74 | 0.37 ± 0.19 | 0.38 ± 0.15 | 0.38 ± 0.18 |  |  |  |
| **PMA-treated 2Y** | 31 | 0.38 ± 0.18 | 0.41 ± 0.14 | 0.39 ± 0.17 | 0.37 ± 0.19 |  |  |
| **PMA-treated 3Y** | 31 | 0.37 ± 0.20 | 0.32 ± 0.10 | 0.37 ± 0.18 | 0.33 ± 0.14 |  |  |
| **PMA-treated – 2Y+ 3Y** | 62 | 0.38 ± 0.19 | 0.36 ± 0.13 | 0.38 ± 0.17 | 0.35 ± 0.16 |  |  |
| **PMA-treated 3Y** | 29 | 0.38 ± 0.18 | 0.41 ± 0.14 | 0.40 ± 0.17 | 0.38 ± 0.19 | 0.33 ± 0.14 |  |
| **PMA-treated 4Y** | 26 | 0.39 ± 0.22 | 0.34 ± 0.15 | 0.37 ± 0.19 | 0.31 ± 0.11 | 0.32 ± 0.12 |  |
| **PMA-treated 3Y + 4Y** | 55 | 0.38 ± 0.20 | 0.37 ± 0.15 | 0.38 ± 0.18 | 0.35 ± 0.16 | 0.33 ± 0.13 |  |
| **PMA-treated 4Y** | 29 | 0.37 ± 0.18 | 0.41 ± 0.14 | 0.39 ± 0.17 | 0.38 ± 0.19 | 0.33 ± 0.14 | 0.35 ± 0.16 |
| **PMA-treated 5Y** | 26 | 0.37 ± 0.22 | 0.31 ± 0.10 | 0.34 ± 0.15 | 0.31 ± 0.11 | 0.32 ± 0.12 | 0.29 ± 0.10 |
| **PMA-treated – final (4Y+ 5Y)** | 55 | 0.37 ± 0.20 | 0.36 ± 0.13 | 0.36 ± 0.16 | 0.35 ± 0.16 | 0.33 ± 0.13 | 0.32 ± 0.13 |

| **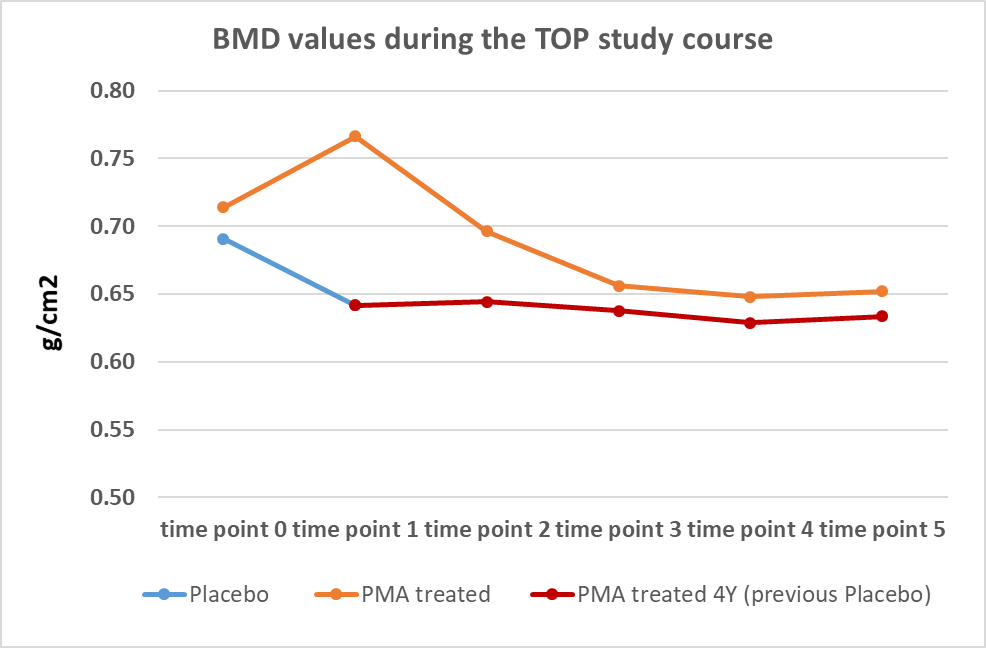** |
| --- |
| **(A)** |
| **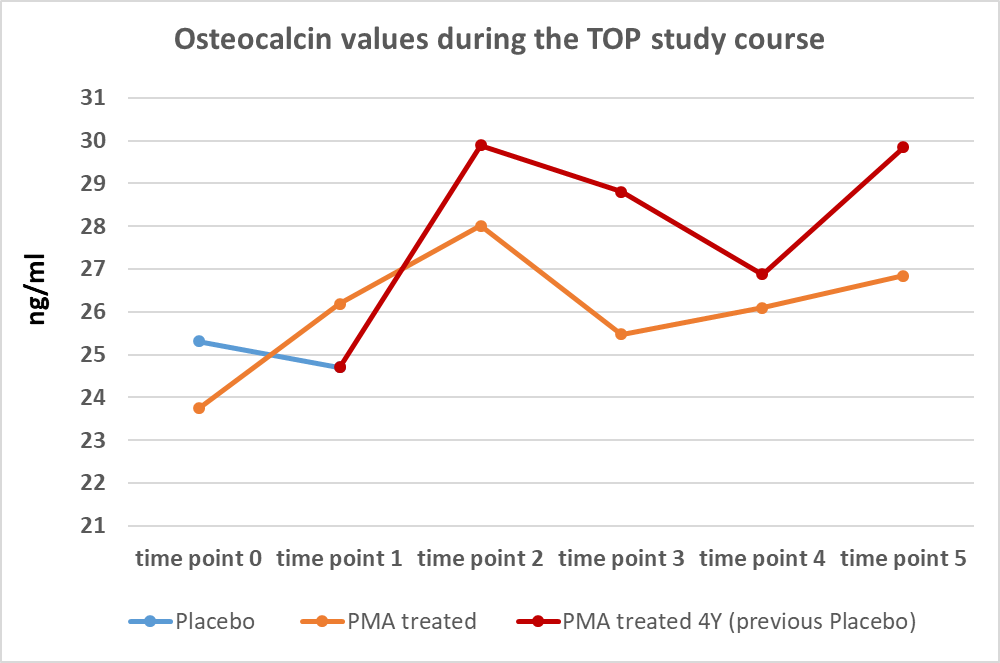** |
| **(B)** |
| **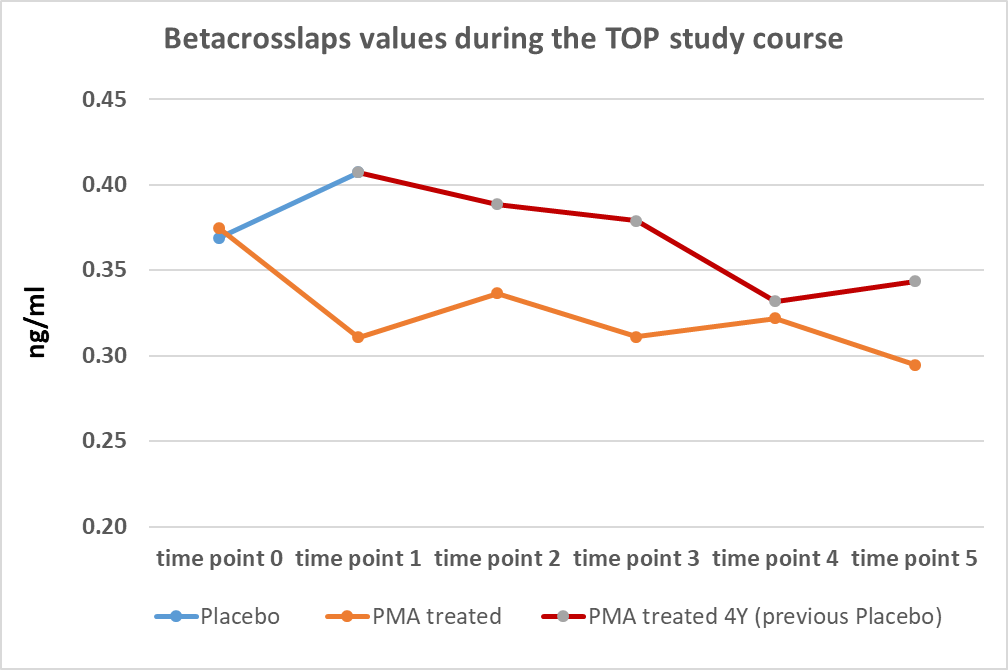** |
| **(C)** |
| **Supplementary figure 1. Changes in BMD (panel A), osteocalcin (panel B) and beta-cross laps (panel C) values from the beginning of the study (time point 0) until the end of study (time point 5). A separate representation is given for the patients enrolled as Placebo group at time point 0 (later treated with PMA-zeolite from time point 1 – 5; bue line) and enrolled patients treated with PMA-zeolite from the beginning of the study (time point 0 – 5).** |

# Analysis of diabetic patients’ subgroup enrolled in the TOP study

The diabetic subgroup comprised 8 patients and showed different behavior during PMA-treatment that is explained in Supplementary table 2.

**Supplementary table 3. Bone parameters for Placebo time point 1 and PMA-treated patients for 1-5 years (1Y – 5Y), compared between the non-diabetic and diabetic patients’ subgroups. Statistically relative differences at p<0.05 are marked with an asterisk.**

| **Parameter** | **Placebo (at the end of 1st study year)** | | | | | | **PMA-treated patients 2Y** | | | | | **PMA-treated patients 3Y** | | |
| --- | --- | --- | --- | --- | --- | --- | --- | --- | --- | --- | --- | --- | --- | --- |
|  | **Non-diabetics (N=32)** | | **Diabetics (N=8)** | | ***p*** | | **Non-diabetics (N=26)** | | **Diabetics (N=5)** | | ***p*** | **Non-diabetics (N=23)** | **Diabetics (N=8)** | ***p*** |
| **BMD at the beginning of study** | 0.681 ± 0.107 | | 0.732 ± 0.107 | | 0.267 | | 0.626 ± 0.189 | | 0.586 ± 0.061 | | 0.470 | 0.625 ± 0.097 | 0.673 ± 0.161 | 0.315 |
| **BMD at the end of study** | 0.651 ± 0.100 | | 0.704 ± 0.132 | | 0.216 | | 0.645 ± 0.124 | | 0.609 ± 0.071 | | 0.534 | 0.636 ± 0.114 | 0.663 ± 0.159 | 0.615 |
| **Relative Δ BMD / %** | -4.2 ± 4.4 | | -3.6 ± 3.0 | | 0.713 | | +2.9 ± 3.9 | | +3.7 ± 3.7 | | 0.664 | +0.9 ± 5.5 | -1.6 ± 3.7 | 0.238 |
| **Osteocalcin at the beginning of study** | 26.6 ± 8.5 | | 17.6 ± 4.9 | | **0.007*** | | 25.6 ± 7.1 | | 17.5 ± 3.6 | | **0.020*** | 28.9 ± 9.9 | 20.9 ± 10.7 | 0.064 |
| **Osteocalcin at the end of study** | 25.4 ± 6.6 | | 17.8 ± 4.1 | | **0.004*** | | 29.9 ± 11.0 | | 18.7 ± 5.1 | | **0.035*** | 26.8 ± 7.4 | 21.0 ± 11.1 | 0.105 |
| **Relative Δ Osteocalcin/ %** | -1.0 ± 18.9 | | + 3.6 ± 14.8 | | 0.533 | | +17.5 ± 3.6 | | +5.6 ± 12.6 | | 0.444 | -4.1 ± 18.7 | +1.2 ± 19.7 | 0.502 |
| **Betacrosslaps at the beginning of study** | 0.39 ± 0.18 | | 0.25 ± 0.12 | | **0.046*** | | 0.41 ± 0.15 | | 0.28 ± 0.07 | | 0.066 | 0.36 ± 0.12 | 0.26 ± 0.08 | **0.034*** |
| **Betacrosslaps at the end of study** | 0.41 ± 0.14 | | 0.30 ± 0.45 | | **0.044*** | | 0.39 ± 0.20 | | 0.30 ± 0.09 | | 0.325 | 0.35 ± 0.15 | 0.28 ± 0.08 | 0.226 |
| **Relative Δ Betacrosslaps/ %** | 16.0 ± 40.0 | | + 44.5 ± 92.7 | | 0.188 | | -6.4 ± 36.9 | | +6.7 ± 29.6 | | 0.461 | +4.3 ± 48.2 | +12.8 ± 24.2 | 0.639 |
| **Parameter** | **PMA-treated patients 4Y** | | | | | **PMA-treated patients 5Y** | | | | | |  |  |  |
|  | **Non-diabetics (N=23)** | **Diabetics (N=7)** | | ***p*** | | **Non-diabetics (N=19)** | | **Diabetics (N=7)** | | ***p*** | |  |  |  |
| **BMD at the beginning of study** | 0.674 ± 0.093 | 0.745 ± 0.045 | | 0.121 | | 0.685 ± 0.092 | | 0.792 ± 0.063 | | **0.009*** | |  |  |  |
| **BMD at the end of study** | 0.627 ± 0.094 | 0.703 ± 0.119 | | 0.103 | | 0.631 ± 0.095 | | 0.709 ± 0.121 | | 0.101 | |  |  |  |
| **Relative Δ BMD / %** | -7.4 ± 14.7 | -10.8 ± 15.2 | | 0.605 | | -6.9 ± 15.2 | | -10.2 ± 15.4 | | 0.623 | |  |  |  |
| **Osteocalcin at the beginning of study** | 26.9 ± 11.1 | 15.8 ± 6.5 | | 0.013* | | 26.3 ± 9.4 | | 16.9 ± 6.2 | | **0.024*** | |  |  |  |
| **Osteocalcin at the end of study** | 27.7 ± 7.6 | 21.6 ± 11.8 | | 0.130 | | 28.2 ± 4.9 | | 23.1 ± 10.2 | | 0.093 | |  |  |  |
| **Relative Δ Osteocalcin/ %** | +8.8 ± 22.3 | +27.7 ± 41.2 | | 0.144 | | +14.6 ± 28.3 | | +38.8 ± 33.5 | | 0.078 | |  |  |  |
| **Betacrosslaps at the beginning of study** | 0.42 ± 0.23 | 0.28 ± 0.23 | | 0.111 | | 0.40 ± 0.22 | | 0.29 ± 0.17 | | 0.255 | |  |  |  |
| **Betacrosslaps at the end of study** | 0.36 ± 0.12 | 0.23 ± 0.07 | | 0.016* | | 0.31 ± 0.09 | | 0.25 ± 0.10 | | 0.205 | |  |  |  |
| **Relative Δ Betacrosslaps/ %** | 7.1 ± 21.9 | -5.7 ± 31.6 | | 0.561 | | -5.3 ± 55.1 | | -1.8 ± 47.2 | | 0.882 | |  |  |  |

While comparing the subgroups of diabetic patients with non-diabetic patients treated with PMA, it is clear that the effects of PMA on surrogate bone parameters BMD, osteocalcin and betacross laps were lower and statistically relevant for osteocalcin (0.024*) and higher for BMD (0.009*).
